# Current Role of Computed Tomography in the Evaluation of Acute Coronary Syndrome

**DOI:** 10.3390/diagnostics11020266

**Published:** 2021-02-09

**Authors:** Min Ji Son, Seung Min Yoo, Dongjun Lee, Hwa Yeon Lee, In Sup Song, Eun Ju Chun, Charles S White

**Affiliations:** 1 Department of Radiology, CHA University Bundang Medical Center, Bundang 13497, Korea; a176008@chamc.co.kr; 2 Military Service in Korean Army, Hongcheon 25117, Korea; sirious0416@naver.com; 3 Smile Radiologic Clinic, Seoul 33066, Korea; hynlee1@hanmail.net; 4 Department of Radiology, Chun Ju Jesus General Hospital, Chun Ju 54987, Korea; issong1@nate.com; 5 Department of Radiology, Seoul National University Bundang Medical Center, Seongnam 13620, Korea; drejchun@hanmail.net; 6 Department of Radiology, University of Maryland, Baltimore, MD 21201, USA; cwhite@umm.edu

**Keywords:** computed tomography, coronary CT angiography, acute coronary syndrome, acute chest pain

## Abstract

This review article provides an overview regarding the role of computed tomography (CT) in the evaluation of acute chest pain (ACP) in the emergency department (ED), focusing on characteristic CT findings.

## 1. Introduction

Acute chest pain (ACP) is the second most common presentation in the emergency department (ED) [1]. As missed diagnosis of acute coronary syndrome (ACS) is frequently associated with a poor clinical outcome, immediate diagnosis and exclusion of ACS are of paramount importance. In patients with a high risk of ACS, prompt invasive coronary angiography and/or revascularization should be performed without noninvasive imaging in order to salvage viable myocardium. In contrast, in patients with a low to moderate risk of ACS, standard assessment includes symptoms and signs, physical examination, serial ECGs, and cardiac troponins, followed by optional functional testing (i.e., exercise ECG, rest and/or stress perfusion imaging, or stress echocardiography) [2,3]. However, this standard protocol may be associated with a lengthy stay in the ED or coronary care unit awaiting ECG or troponin changes or stress testing, leading to ED overcrowding and increased costs. Notably, cutting-edge CT (64-slice CT or newer generation CT scanners) has the capability to rapidly and reliably exclude non-ST-segment elevation ACS by directly visualizing the coronary arterial wall [2,3]. In addition, the recent approval of high-sensitivity troponins (hs-Tn) has overcome previous shortcomings of conventional troponin assays by allowing the detection of acute myocardial infarction (AMI) at an earlier stage. [4,5]. Thus, there may be uncertainty among ED physicians and cardiologists regarding the best option (i.e., anatomic (CT) versus hs-Tn with functional testing) for evaluating ED chest pain [6].

This review article summarizes insights from multiple studies regarding the strengths and shortcomings of coronary CTA over a traditional management protocol of ACP and provides an overview of basic CT techniques and characteristic CT findings of ACS.

## 2. Insights from Recent Randomized Controlled Trials Regarding Coronary CTA in the Evaluation of Acute Chest Pain

### 2.1. Randomized Controlled Trials Prior to the Era of High-Sensitivity Troponin

The ROMICAT II and ACRIN trials reported very high negative predictive values for coronary CTA in excluding > 50% coronary stenosis in ED settings, demonstrating that this approach can lead to a more rapid and safer discharge from the ED (47% vs. 12%, *p* < 0.001, and 49.6% vs. 22.7%, *p* < 0.001, respectively), a shorter the ED stay (8.6 h vs. 26.7 h, *p* < 0.001, and 18.0 h vs. 24.8 h, *p* < 0.001, respectively), and improved ED cost-effectiveness compared to the standard assessment [7,8]. However, there was no definite short-term outcome benefit in the CT arm compared to standard care in these trials. In contrast, the CATCH trial demonstrated a midterm outcome benefit (i.e., major adverse cardiac events, including cardiac death, AMI, unstable angina, and symptom-driven revascularization; *p* = 0.04; HR: 0.36 (95% CI: 0.16 to 0.95)) in the CT arm over the standard assessment [9]. The contradictory results between the CATCH (Denmark) and other trials may be caused by the time difference in defining outcome and different referral patterns compared to the US. The outcome was measured as a short-term parameter (<30 days) in the ROMICAT II and ACRIN trials versus a midterm parameter in the CATCH trial (median, 18.7 months). The favorable results in CT outcome obtained from the CATCH trial may reflect the ability of CT to precisely identify obstructive and nonobstructive coronary artery disease. The identification of obstructive stenosis may have resulted in the better use of invasive coronary angiography and revascularization, leading to a decrease in episodes of unstable angina in the midterm to a greater extent than in the short term. Moreover, increased identification of nonobstructive coronary artery stenosis in the CT arm may have resulted in the greater use of statins [10]. However, there remains debate as to whether using a CT protocol causes increased downstream testing as coronary CTA has a nonnegligible false-positive rate, mainly due to blooming artifacts of calcified plaque. According to a meta-analysis of the outcomes of randomized controlled trials of coronary CTA in the ED, the use of coronary CTA was associated with decreased ED cost and length of stay but increased rates of coronary angiography and revascularization [11]. In summary, the use of a CT protocol in the ACP setting is associated with a reduced ED stay and/or costs compared to standard care with conventional troponins. However, further randomized trials are required to address whether there is an outcome benefit and any effects on downstream testing.

### 2.2. Randomized Controlled Studies in the Era of High-Sensitivity Troponin

The ROMICAT II, ACRIN, and CATCH trials were performed prior to the approval of hs-Tn. In contrast, the BEACON trial used hs-Tn and showed that there was no difference in the length of ED stay (both groups = 6.3 h) between the CT and standard assessment groups [12]. These results may portend the decreased utility of coronary CTA in patients with a low risk of ACS and a normal hs-Tn level. In addition, the BEACON trial did not demonstrate a 30-day outcome benefit over standard care (CT and standard arm, 10% vs. 9%, respectively, >0.05). The negative result regarding a short-term outcome benefit is expected considering that a majority of the enrolled patients had a low pretest probability (84%) in the trial [12]. However, it is unclear whether the identification of obstructive and nonobstructive coronary stenosis by CT might result in an outcome benefit during a longer follow-up in patients with an intermediate risk of ACS. It is hoped that the ongoing RAPID-CTCA trials will answer this question in the near future. In summary, the BEACON trial suggested that CT does not reduce ED stay length in the era of hs-Tn compared to standard care [13]. Thus, it is expected that hs-Tn may be useful to exclude ACS in a manner similar to the role of D-dimer in the evaluation of patients at low risk of pulmonary embolism in the ED. In addition, it remains unclear whether the use of a CT-driven protocol is beneficial in terms of outcome over standard assessment.

## 3. What Is the Proper Indication for Coronary CT in the Evaluation of Acute Chest Pain?

### 3.1. Definition of Pretest Probability for Acute Chest Pain

Some clinical features are known to be valuable in differentiating ACP caused by ACS from other etiologies. For instance, pain aggravated by deep inspiration or pain reproducible on palpation are often caused by diseases other than ACS. Pain caused by ACS is often characterized by substernal heaviness or squeezing in nature, lasting more than 30 min and/or radiating to the neck or arms. However, precise discrimination of ACS from the other causes of ACP is often difficult based merely on the assessment of the patient’s symptoms and signs [14]. There are multiple scoring systems used to predict the pretest probability of ACS in patients with ACP (e.g., TIMI, GRACE, HEART, EDACS score, and Diamond–Forrester criteria) [15,16,17,18,19]. Indeed, the previous randomized controlled trials used different tools in their studies. For instance, the ROMICAT II and BEACON trials used TIMI and TIMI and GRACE scores, respectively, while the CATCH trial used both the Diamond–Forrester criteria and TIMI risk score to assess pretest probability. However, the Diamond–Forrester criteria consist of patient age, sex, and the presence or absence of typical angina. The definition of typical angina should have all three characteristic components (substernal squeezing pain, aggravating and relieving factors). However, mitigating and/or aggravating factors are often absent in patients with ACS, rendering the scoring system less than optimal. Thus, application of the Diamond–Forrester criteria may be more suitable for patients with a concern for stable angina. Although each scoring system has different components, atypical or typical chest pain accompanied by a nondiagnostic ECG and normal initial troponins is commonly classified as a low or intermediate risk of ACS. Tatum et al. defined an intermediate risk of ACS as typical ACP lacking objective evidence of ACS (i.e., substernal ACP with squeezing or heaviness lasting >30 min but with no ischemic ECG abnormalities and troponin elevation). In addition, a low risk of ACS was defined as typical ACP lasting <30 min or atypical ACP lasting >30 min unaccompanied by ECG or troponin abnormalities [20]. The current consensus (Table 1) is that coronary CTA should be reserved for patients with an intermediate or low risk of ACS [21,22]. However, in the future, the role for coronary CTA may be more limited in the subgroup with normal hs-Tn and a low risk of ACS. Intermediate-risk patients with typical chest pain lasting >30 min but normal ECG and hs-Tn may be deemed suitable for coronary CTA in the future. Notably, there may be increasing subgroups with a mild or nonspecific increase in the level of hs-Tn that will be included in the indications for coronary CTA, a cohort in which conventional troponins level would presumably have been normal.

### 3.2. Contraindications to Coronary CTA

Several patient-related conditions may preclude coronary CTA examination. These include hemodynamic instability, renal failure, known coronary artery disease, pregnancy, inability to control heart rate as can occur with arrhythmia, and a history of an allergic reaction to contrast materials.

## 4. Basic CT Techniques and Characteristic Coronary CT Findings of Acute Coronary Syndrome

### 4.1. Basics of Coronary CT Techniques

**Figure 1:**
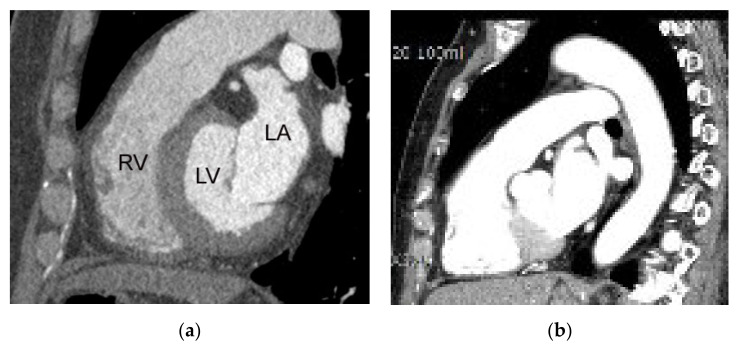
Typical z-axis coverage of dedicated coronary CTA versus triple-rule-out. (**a**) Z-axis coverage of dedicated coronary CTA includes the lower two-thirds of the entire chest. (**b**) The entire chest constitutes the z-axis coverage of the triple-rule-out CT. RV, LA, and LV indicate right ventricle, left atrium, and left ventricle, respectively.

To achieve sufficient image quality during coronary CTA, heart rate control should be optimized using beta-blockers. The target heart rate is different depending on the specific CT machine. In general, single-source and dual-source CT require a rate <60 and <70 bpm/min, respectively, to minimize coronary motion artifacts. In addition, sublingual nitroglycerin should be administered to dilate the coronary arteries, provided there is no contraindication such as hypotension or ongoing use of Sildenafil. In contrast to non-ECG gated chest CT, coronary CTA is inevitably associated with a higher radiation exposure due to the low pitch mode used for ECG gating, especially with single-source CT. Thus, measures to reduce radiation exposure should be employed, especially in younger female patients. There are two basic options available to acquire coronary CTA, retrospective and prospective ECG gating. The former has greater radiation exposure as images are obtained throughout the cardiac cycle but has the advantage that it enables the acquisition of a cine loop, permitting assessment of left ventricular function [3]. Another important issue is whether a triple-rule-out examination is appropriate in patients with nonspecific ACP. Triple rule-out may be performed in order to simultaneously exclude aortic dissection, pulmonary embolism, and ACS in patients with nonspecific ACP. The field of view for dedicated coronary CTA typically includes the lower two-thirds of the chest. With this field of view, most aortic dissections and pulmonary emboli are demonstrated on dedicated coronary CTA [23]. Thus, a triple-rule-out should be avoided in younger female patients with nonspecific ACP due to the greater radiation exposure caused by wider z-axis coverage (Figure 1).

### 4.2. Coronary Artery Abnormalities of Acute Coronary Syndrome

#### 4.2.1. How to Identify Coronary Abnormalities on Coronary CTA

##### Culprit Plaque of Type 1 Acute Myocardial Infarction 

**Figure 2:**
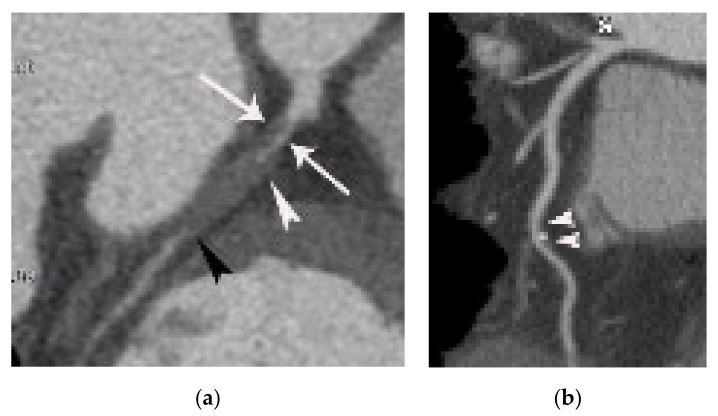
Difference between culprit (**a**) and nonculprit plaque (**b**) on coronary CTA. (**a**) Note the total occlusion (arrowheads) by noncalcified plaque with vulnerable CT features (positive remodeling and napkin ring sign appearance (arrows)) in the proximal left circumflex artery in a patient with AMI, showing it to be the culprit lesion. (**b**) In contrast, <50% stenosis with mixed plaque without vulnerable CT features (arrowheads) is noted in the mid left anterior descending coronary artery in a nonculprit lesion.

A Type 1 AMI is initiated by vulnerable plaque rupture resulting in obstructive coronary stenosis or occlusion. Thus, a culprit lesion is often characterized by a ≥70% stenosis with mixed or noncalcified plaque accompanied by positive remodeling, low-attenuation plaque, the napkin ring sign, and spotty calcification (<3 mm in diameter) on coronary CTA (Figure 2). Positive remodeling is defined as the adaptive outward growth of atherosclerotic plaque with a remodeling index > 1.1 (the remodeling index = coronary artery diameter at the level of a plaque/coronary artery diameter at a nearby reference level without plaque). Low-attenuation plaque on CT is often defined by Hounsfield units (HU) < 30, indicating a lipid core [24,25,26]. However, measurement of a low-attenuation plaque may be imprecise due to partial volume averaging depending on the amount of intraluminal coronary enhancement on CT, rendering this finding less specific. The napkin ring sign is defined by a CT plaque demonstrating inner lower and outer higher attenuation (i.e., inhomogeneous plaque with lower internal attenuation) [27]. A thin-cap fibroatheroma is a major feature of vulnerable plaque on microscopic examination. Because the thickness of the thin-cap fibroatheroma is <65 μm, it cannot be directly visualized on CT due to limits of spatial resolution [28]. A thin-cap fibroatheroma can be presumed to be present on CT based on the identification of the napkin ring sign. However, even the combination of obstructive stenosis and vulnerable CT features does not necessarily indicate a culprit lesion of ACS.

**Figure 3:**
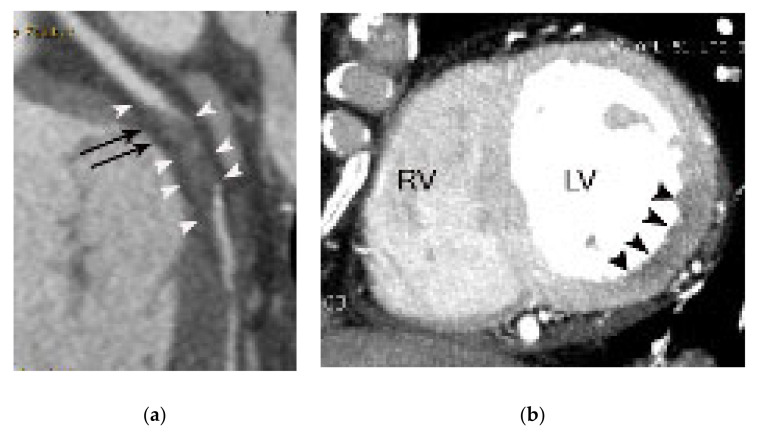
Fat stranding in a 75-year-old male patient with acute myocardial infarction referable to the left circumflex coronary artery. (**a**) Total occlusion (arrowheads) is noted in the proximal portion of the distal left circumflex coronary artery. Note the positive remodeling and subtle irregular lines (arrows) at the epicardial fat interface near the occluded coronary wall, suggesting epicardial fat stranding. (**b**) Subendocardial hypoattenuation (arrowheads) is demonstrated in the territory of the left ventricle on a short-axis view, consistent with myocardial necrosis. RV and LV indicate right and left ventricle, respectively.

This raises the issue of how to discriminate a culprit lesion from an innocent bystander lesion with vulnerable CT features. During an episode of ACS, two main changes occur within a precursor lesion with vulnerable features [26]. The first alteration is the tearing of the thin cap with the expulsion of the lipid core of plaque into the bloodstream. The second change is sudden thrombus formation within the ruptured coronary segment. Chun et al. [26] reported that there was no difference in the prevalence of the CT signs of low-attenuation plaque and the napkin ring sign among precursor and culprit lesions in patients with ACS. Thus, they assumed that the first changes may be subtle and likely below the spatial resolution of CT. In contrast, total occlusion suggesting thrombus formation, and myocardial hypoattenuation suggesting myocardial necrosis is more common in a culprit lesion compared to a precursor lesion on CT [26]. The coronary occlusion is due to thrombosis after the episode of ACS. Several points (Figure 3) are helpful to differentiate a culprit lesion from a nonculprit obstructive coronary stenosis on CT, including (1) vulnerable plaque features, (2) myocardial hypoattenuation or wall motion abnormality, and (3) epicardial fat stranding.

##### Culprit Plaque of Type 2 Acute Myocardial Infarction

**Figure 4:**
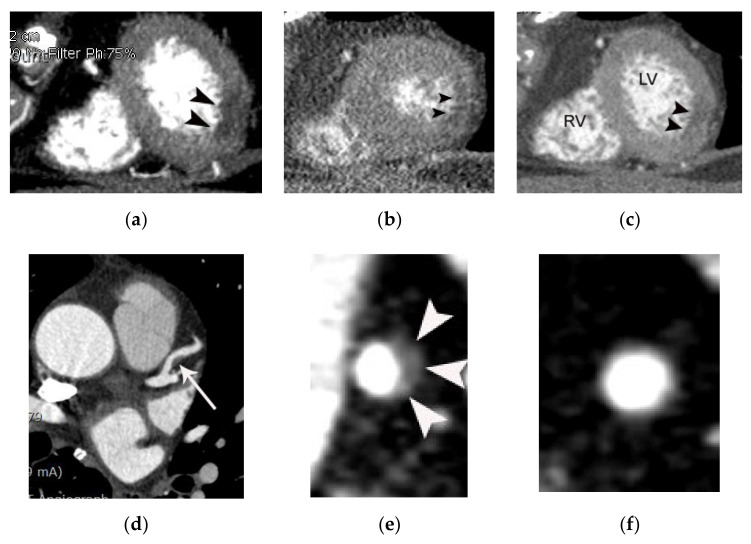
A case of myocardial infarction with nonobstructive coronary arteries (MINOCA) in a 66-year-old female patient. The patient presented to the emergency department (ED) with acute chest pain. Focal hypoattenuation (arrowheads in (**a**)) in the lateral wall of the apex of the left ventricle is noted on a CT short-axis view. There is akinesia in the same location on a short-axis systolic image (**b**). Note the absence of wall thickening in this area on the systolic image compared to the diastolic image (**b**,**c**). However, the coronary arteries were normal other than nonobstructive coronary stenosis (arrow in (**d**)) with noncalcified plaque and positive remodeling in the proximal left anterior descending coronary artery on an axial coronary CT image (**d**). Note the noncalcified plaque with positive remodeling on an en face view (arrowheads in (**e**)) compared to the normal reference level (**f**). Subsequent coronary angiography confirms the CT findings (not shown). RV and LV indicate right and left ventricle, respectively.

Type 2 AMI is caused by a mismatch of blood supply and demand. Type 2 AMI is often referred to as myocardial infarction with nonobstructive coronary arteries (MINOCA) (Figure 4) [29]. MINOCA may be due to prolonged coronary spasm, spontaneous thrombolysis, and coronary embolism. Based on the theory of spontaneous thrombolysis in the MINOCA, an initial event assumed to be similar to that of typical vulnerable plaque rupture results in obstructive coronary stenosis. However, exaggerated spontaneous thrombolysis may be endogenously initiated in patients with MINOCA, leading to evolution to nonobstructive coronary stenosis. The coronary spasm in MINOCA may extend for a sufficient length of time to result in myocardium necrosis. Coronary angiography or CT may be normal or demonstrate < 50% stenosis after the spasm is relieved [29].

##### Culprit Plaque in Unstable Angina

**Figure 5:**
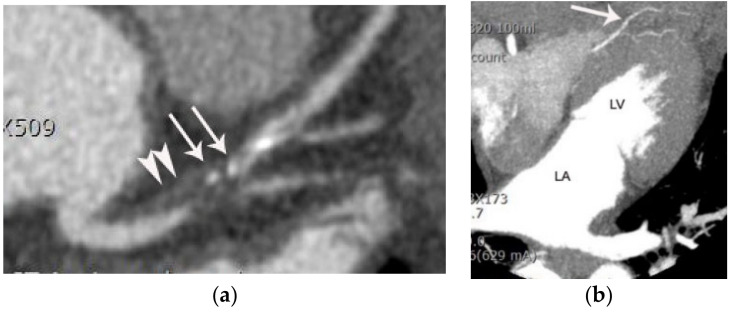
A case of unstable angina in a 61-year-old male patient with crescendo angina. (**a**) Curved multiplanar reformatted image shows near-total occlusion in the distal left main coronary artery. Note the napkin ring sign (arrowheads), positive remodeling, and spotty calcifications (arrows). However, there is no myocardial hypoattenuation in the left ventricle on a four-chamber image. Note a collateral artery (arrow in (**b**)) from the posterior descending coronary artery coursing to the distal left anterior descending coronary artery. LV and LA indicate left ventricle and atrium, respectively.

ACS consists of ST-elevated AMI, non-ST-elevated AMI, and unstable angina. The CT features of unstable angina (Figure 5) are similar to those of type 1 AMI but lack the wall motion abnormality and myocardial hypoattenuation in the affected arterial territory as there is no myocardial necrosis.

### 4.3. Epicardial Fat Abnormality of Acute Coronary Syndrome

Plaque inflammation is an important factor underlying ACS (i.e., vulnerable plaque rupture) resulting in inflammatory thinning of the fibrous cap. Previous studies have indicated that there is inflammatory cell infiltration in the media, adventitia, and epicardial fat as well as intima in patients with ACS [30,31,32]. Thus, epicardial inflammation may also be demonstrated on coronary CT as epicardial fat stranding (Figure 3). In patients without ACS, a sharp demarcation between the coronary artery wall and epicardial fat is present, even in plaques with positive remodeling. In contrast, in some patients with ACS (<50%), this sharp demarcation may be lost resulting in irregularity and obscuration of epicardial fat [33].

### 4.4. Myocardial Abnormalities of Acute Coronary Syndrome

**Figure 6:**
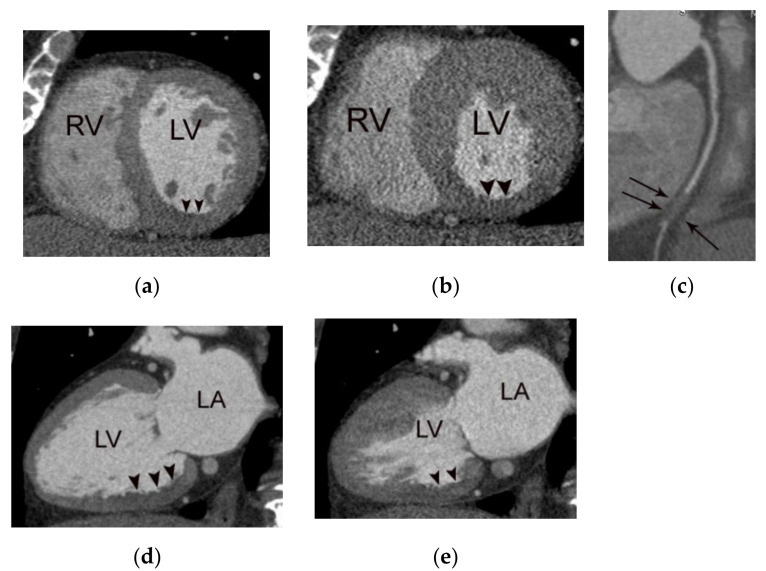
A case of acute myocardial infarction in a 60-year-old male patient. A. Note the akinesia (arrowheads) in the inferior wall of the mid to basal left ventricle in diastolic (**a**,**d**) and systolic images (**b**,**e**). Note the total occlusion in the distal right coronary artery (arrows in (**c**)) with positive remodeling and fat stranding, resulting in an irregular interface between the coronary wall and epicardial fat. RV, LA, and LV indicate right ventricle, left atrium, and left ventricle, respectively.

**Figure 7:**
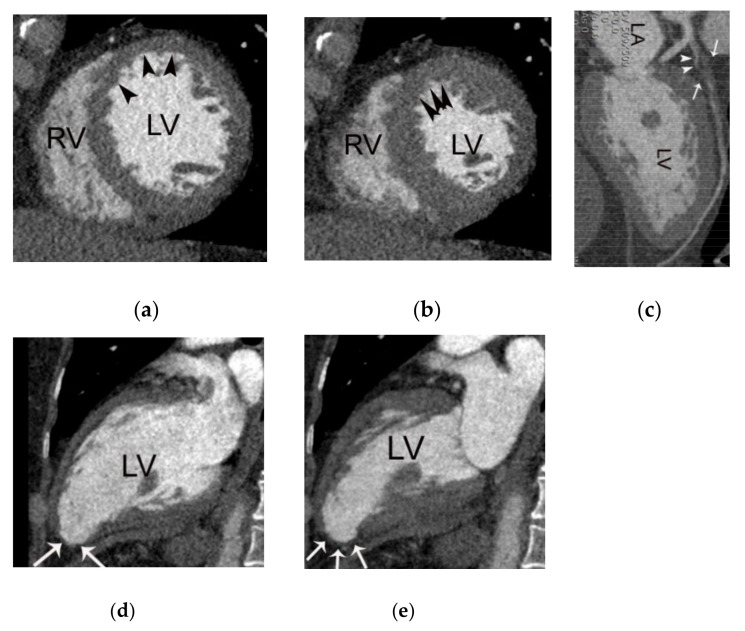
A case of acute myocardial infarction in a 61-year-old male patient. (**a**) Note the akinesia in the left anterior descending coronary artery territory in diastolic (**a**,**d**) and systolic images (**b**,**e**) (arrowheads or arrows). Note also the total occlusion of the proximal left anterior descending coronary artery by noncalcified plaque (arrows in (**c**)) with positive remodeling, low-attenuation plaque, and a napkin ring sign (arrowheads in (**c**)). RV, LA, and LV indicate right ventricle, left atrium, and left ventricle, respectively.

**Figure 8:**
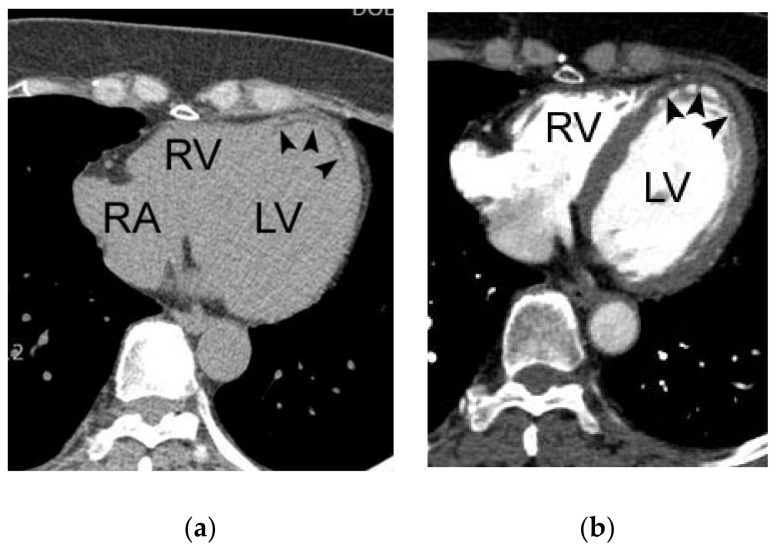
A case of chronic myocardial infarction in the left anterior descending coronary artery in a 63-year-old female patient. Note the subendocardial fat and myocardial thinning in the left ventricular apex (arrowheads in (**a**,**b**)). RV, RA, and LV indicate right ventricle, right atrium, and left ventricle, respectively.

Evaluation for wall motion abnormalities and hypoattenuation in the left ventricle should be performed in all patients with suspicion for ACS. Notably, identification of wall motion abnormality or hypoattenuation along a specific coronary artery territory is valuable in cases with nondiagnostic coronary artery findings on CT due to coronary motion or blooming artifact [34]. It is important to understand the typical appearance of an arterial territory on a short-axis view (Figure 6 and Figure 7) as its evaluation can enhance diagnostic confidence for AMI if there is an obstructive coronary stenosis with vulnerable plaque features in a matching coronary artery territory. Subendocardial hypoattenuation may be present in many cases with AMI on CT. The HU measurement within the area demonstrating hypoattenuation is often less than half that of an area with normal attenuation [35,36]. Transmural hypoattenuation is quite rare as coronary CTA is not performed in most patients with ST-elevated AMI. However, subendocardial hypoattenuation is often demonstrated in patients with non-ST-elevated AMI, suggesting myocardial necrosis and portending a worse prognosis. Hypoattenuation caused by AMI should be differentiated from artifact and chronic myocardial infarction (CMI) on CT. Cases with CMI (Figure 8) have fat attenuation on precontrast CT and are accompanied by myocardial thinning, while there is an absence of thinning in cases with AMI (Figure 6 and Figure 7). Hypoattenuation secondary to motion or beam hardening artifact typically does not conform to a specific vascular territory.

### 4.5. Complications of Acute Myocardial Infarction

**Figure 9:**
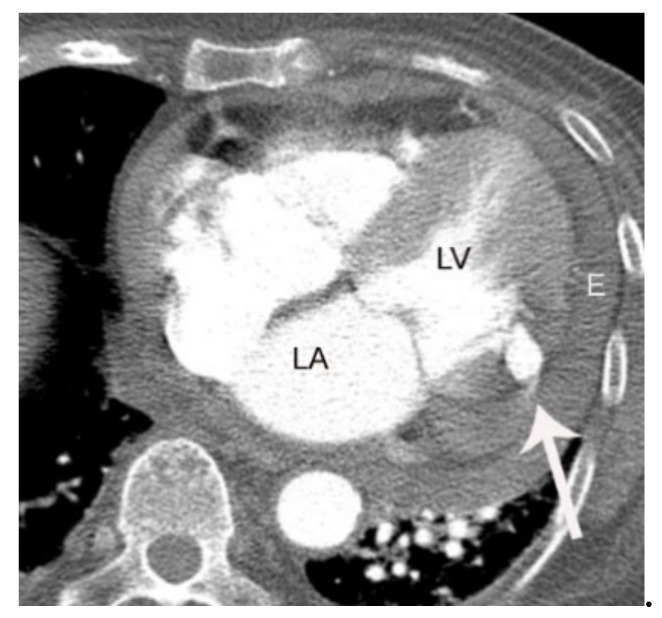
A case of left ventricular free-wall rupture in an 84-year-old male patient who presented with cardiogenic shock. Note the rupture site (arrow) and pericardial hematoma (E). LA and LV indicate left atrium and ventricle, respectively.

Complications of AMI include pulmonary edema, acute mitral regurgitation, ventricular septal defect, and free-wall rupture [36]. Acute mitral regurgitation can occur if AMI affecting the left circumflex coronary artery results in papillary muscle necrosis [37]. The posteromedial papillary muscle is often involved in such cases due to its solitary blood supply. Free-wall rupture caused by AMI is a rare life-threatening complication presenting with shock (Figure 9) [38].

### 4.6. Which Vulnerable Plaque Will Eventually Lead to Acute Coronary Syndrome?

**Figure 10:**
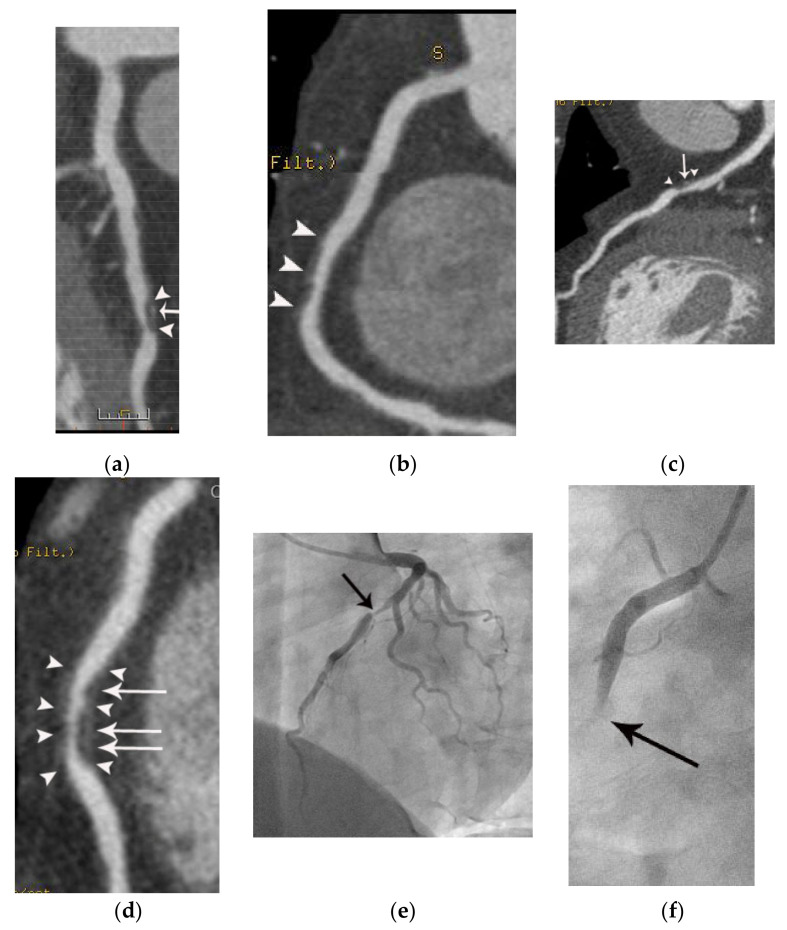
A 46-year-old man presenting with ACS. Coronary CTA was obtained due to multiple cardiac risk factors. Approximately 70% stenoses with mixed plaque (**a**) and 40–50% stenosis with noncalcified plaque (**b**) are noted in the mid left anterior descending and mid right coronary artery, respectively. At the 12-month follow-up coronary CTA, the stenosis in the mid left anterior descending coronary artery shows no definite change (**c**). In contrast, note the progression of plaque (**d**) in the mid right coronary artery in the degree of coronary stenosis (about a 70% stenosis), the increase in the positive remodeling index (arrowheads in (**d**)) and the low-attenuation plaque/napkin ring sign (arrows in (**d**)). Invasive coronary angiography was recommended in this patient, but the patient refused and was lost to follow-up. He presented to the ED with acute chest pain 2 months later. Emergent coronary angiography showed total occlusion indicating a culprit lesion in the same location as the vulnerable plaque in the mid right coronary artery (arrow in (**f**)). In contrast, the stenosis in the proximal middle left anterior descending coronary artery (nonculprit lesion) showed no interval change (arrow in (**e**)).

Not all vulnerable plaques eventually rupture and cause ACS. However, one study indicated that the presence of plaque progression defined as an increasing degree of coronary stenosis or remodeling index, or increasing size of the lipid core on follow-up coronary CT was associated with an increased risk of a future cardiac event [39]. Thus, the progression of vulnerable plaque morphology may be more important in predicting a future episode of ACS than vulnerable plaque morphology visualized on CT at a single point in time (Figure 10).

### 4.7. Alternative Diagnosis

**Figure 11:**
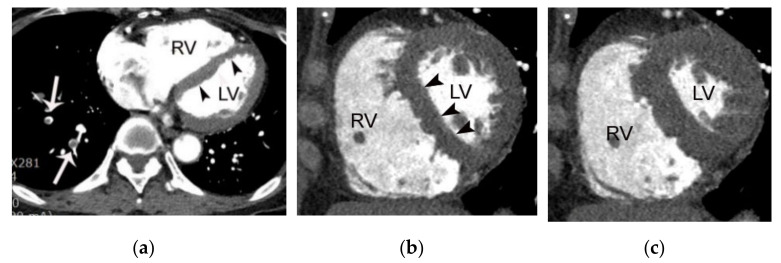
A case of submassive pulmonary embolism in a 66-year-old female patient shown on coronary CTA. (**a**) Multiple pulmonary emboli (arrows) are noted in the right lower lobe on an axial CT image using a wide field of view. Note marked dilatation of the right ventricle and straightening of the interventricular septum (arrowheads) suggesting increased right ventricular pressure. A decrease in the contractile function of the right ventricle can be assessed in this patient due to the use of retrospective ECG gating. Note the lack of change in the right ventricular volume between the diastolic (**b**) and systolic (**c**) short-axis images indicating right ventricular dysfunction. RV and LV indicate right and left ventricle, respectively.

**Figure 12:**
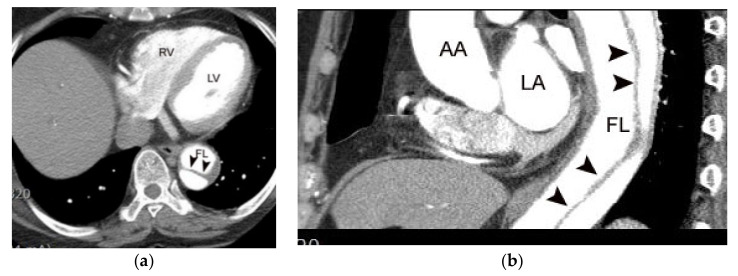
A case of type B aortic dissection identified on coronary CTA. Note the intimal flap within the descending thoracic aorta (arrowheads in (**a**,**b**)) RV, LA, LV, AA, FL indicate right ventricle, left atrium, left ventricle, ascending aorta, and false lumen, respectively.

**Figure 13:**
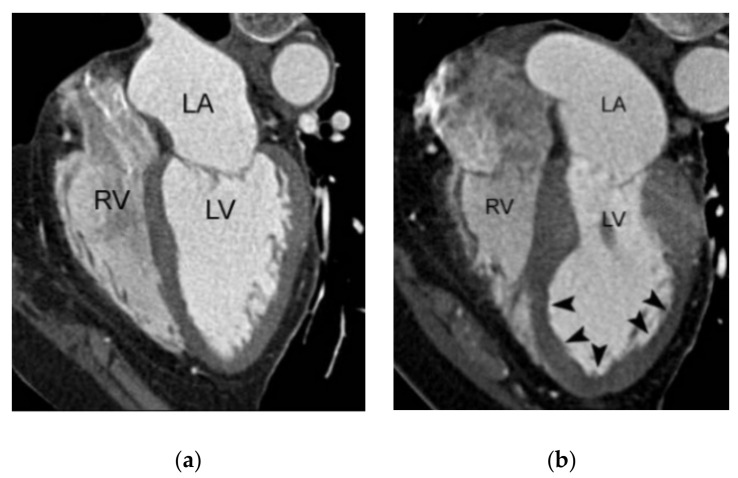
A case of stress-induced cardiomyopathy in a 65-year-old female patient identified on coronary CTA. Typical apical ballooning (arrowheads in (**b**)) is noted on a systolic four-chamber image in comparison to a diastolic image (**a**). There was no obstructive coronary artery stenosis on coronary CTA or invasive coronary angiography (not shown).

**Figure 14:**
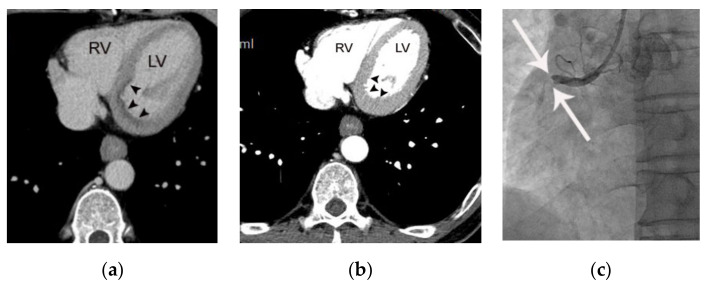
A case of acute myocardial infarction in a 60-year-old male patient with hepatocellular carcinoma identified on non-ECG gated CT. (**a**) Abdomen CT was performed in this patient for routine follow-up of hepatocellular carcinoma. Note the typical subendocardial hypoattenuation without myocardial thinning (arrowheads) in the right coronary artery territory (basal left ventricle). HU of the low and normal attenuation areas in the left ventricle were 45 and 109, respectively. The constellation of CT findings was consistent with AMI in the right coronary artery territory, although there was no corroborating information regarding clinical history, ECG, and troponin level. (**b**) Note the normal attenuation in the same area (arrowheads) on an axial CT image 2 months previously. The ED physician was notified of the CT findings and subsequently confirmed ischemic ECG changes and an elevated troponin level. (**c**) Emergent coronary angiography confirmed total occlusion of the proximal right coronary artery (arrows), and subsequent revascularization was performed.

Clinically important alternative diagnoses (e.g., aortic dissection, pulmonary embolism, or stress-induced cardiomyopathy) can be made on coronary CTA [2]. This is a compelling advantage of anatomical imaging over functional testing (Figure 11, Figure 12, Figure 13 and Figure 14).

### 4.8. Incidental Identification of Acute Myocardial Infarction on Nongated Abdomen or Chest CT

Although nongated chest or abdomen CT is not indicated in the evaluation of ACS, an occasional AMI is incidentally identified on nongated CT (Figure 14). Thus, caution should be exercised to avoid overlooking this important finding, and the myocardium should be inspected as part of all such studies [36,40].

### 4.9. Ability of Coronary CTA to Predict Future Cardiac Event

A previous study indicated that the type (i.e., mixed or noncalcified plaque rather than calcified plaque) and extent of coronary plaque as well as the presence of wall motion abnormalities on the coronary CTA are associated with increased risk of a future cardiac event in the patients presenting with ACP [41]. The potential for coronary CTA to predict future cardiac events may be improved if a better correlation of high-risk plaque can be established with findings on optical coherence tomography [42].

## 5. Future Directions

The lack of functional information is an important shortcoming of coronary CTA. In fact, the application of coronary CTA in the ED may have the potential to increase the use of invasive coronary angiography due to its non-negligible false-positive diagnosis rate. However, recent studies suggest that the shortcoming can be overcome by adding a CT fractional flow reserve to coronary CTA, which has shown favorable results in ED settings as well as in planning for complex percutaneous coronary intervention [43,44]. In addition, ongoing advances in CT hardware and software, including the application of dual-energy CT and artificial intelligence approaches, will likely increase the diagnostic accuracy of CT in ED settings.

## 6. Conclusions

Coronary CTA is an attractive option to exclude ACS in patients with a low to intermediate risk due to its high negative predictive value. A precise understanding of the typical CT findings of ACS can assist interpreting physicians in providing a rapid and precise ACS diagnosis leading to improvement in patient outcome.

## Figures and Tables

**Table 1:** Guidelines for the use of coronary CTA in patients with possible acute coronary syndrome (ACS) presenting without ST elevation.

Indication	
2020 ESC guidelines [21]	Low or intermediate risk of ACS with normal or inconclusive troponin and ECG
	Class I and level of evidence A
2014 AHA/ACC guidelines [22]	Normal ECG, normal cardiac troponin, and no history of coronary artery disease
	Class IIa and level of evidence A

## Dataset License

License under which the dataset is made available (CC0, CC-BY, CC-BY-SA, CC-BY-NC, etc.)
